# Supplementary material for: Mitochondrial and Redox Changes in Periodontitis and Type 2 Diabetes Human Blood Mononuclear Cells
Source: Antioxidants (Basel). 2023 Jan 18;12(2):226. doi: 10.3390/antiox12020226 (PMC9952049; doi:10.3390/antiox12020226)
Supplement: Supplementary file 1 [file antioxidants-12-00226-s001.zip › antioxidants-2151353-supplementary.pdf]

SUPPLEMENTARY MATERIAL

Title: '*Mitochondrial and redox changes in periodontitis and type 2 diabetes human blood mononuclear cells'*

Methods

Analysis of Mitochondrial Oxygen Consumption Rate by Seahorse Analyzer

For oxygen consumption rate (OCR) measurements PBMCs were resuspended in RPMI-1640 R4130 (Sigma-Aldrich, St. Louis, MO, USA) culture medium supplemented with 10 mM Hepes, 12 mM $NaHCO_3$, 2 mM glutamine and 1 mM sodium pyruvate plus heat inactivated 10% FBS. Cells were then plated at 0.5 × 10^6 cells/well in a final volume of about 180 µL in 100 µg/mL poly-D-lysine (molecular weight: 150,000-300,000; Sigma-Aldrich, St. Louis, MO, USA) precoated XF24 microplate wells, and let to adhere for 1.5 - 2h in a humidified incubator chamber with 95% air and 5% CO_2 at 37°C. Cell adherence was checked under an inverted brightfield microscope with 20X magnification. Adhered lymphocytes were then carefully rinsed in order to remove RPMI 1640 culture medium. For this purpose, 100 µL was removed and 100 µL non-buffered DMEM D5030 (Sigma-Aldrich, St. Louis, MO, USA) supplemented with 1.85 g/L de NaCl, 2 g/L glucose, 1 mM sodium pyruvate and 2 mM glutamine, pH 7.4 at 37°C were added to the wells. This procedure was repeated and, after a mild washout, 450 µL of DMEM supplemented as described above was added to the wells. The multi-well plate was then placed for 45 min - 1h in a 37°C-humidified incubator without CO_2, containing 95% air.
Cellular bioenergetics of the isolated cells was determined using the extracellular flux analyzer (Agilent Technologies, Santa Clara, CA, USA), which measures O_2 and protons in the extracellular milieu. This system allows for real time, noninvasive measurement of OCR, which can be correlated with mitochondrial function/oxidative burst. The sequential addition of mitochondria inhibitors oligomycin (Sigma-Aldrich, St. Louis, MO, USA), the uncoupler FCCP (Sigma-Aldrich, St. Louis, MO, USA) and complex I and III inhibitors rotenone (Sigma-Aldrich, St. Louis, MO, USA) plus antimycin A (Sigma-Aldrich, St. Louis, MO, USA), respectively, allows us to determine basal respiration, ATP production, maximal respiration, proton (H^+) leak (indicates passive H^+ leakage across the inner mitochondrial membrane), respiratory resting capacity and non-mitochondrial respiration (defined as oxygen consumption due to a subset of cellular enzymes that consume oxygen in conditions of blockade of cellular respiration achieved by complex I and complex III inhibition), enabling to understand cellular bioenergetics as previously described [21].

Determination of GCL activity

Glutamate-cysteine ligase (GCL) activity was estimated as previously described [25]. In brief, 50 µL of sample was added to 50 µL of pre-warmed reaction buffer (RB) containing (in mM) 400 Tris, 40 ATP (Sigma-Aldrich, St. Louis, MO, USA), 2 EDTA, 20 sodium tetraborate (Santa Cruz Biotechnology, Dallas, TX, USA), 2 L-serine (Sigma-Aldrich, St. Louis, MO, USA), 40 $MgCl_2$ and further incubated 5 min at 37°C. The reaction was then started by adding 50 µL of 2 mM L-cysteine (Sigma-Aldrich, St. Louis, MO, USA) and left 20 min at 37°C. The reaction was stopped by addition of 50 µL of 200 mM 5-sulfosalicylic acid (SSA), vortexing and leaving on ice for another 20 min. Samples were then centrifuged at 2,500 rpm at 4°C for 5 min. A 96-well plate was prepared containing 20 µL of each GSH standard prepared in TES/SB buffer (20 mM Tris, 1 mM EDTA, 250 mM sucrose, 20 mM sodium tetraborate, 2 mM L-serine) plus 50 µL H_2O, 50 µL SSA and 50 µL RB). Following centrifugation, 20 µL of each sample supernatant were added to the plate in triplicate. 180 µL of derivatization solution (NDS: 1.4 parts of 50 mM Tris-HCl, pH 10; 0.2 parts of 0.5 M NaOH; 0.2 parts of 10 mM NDA, diluted in DMSO) were then added to each standard and sample, the plate incubated at room temperature for 30 min in the dark. The analysis was performed at 472 nm excitation and 528 nm emission at 37°C by using a Microplate Spectrofluorometer Gemini EM (Molecular Devices, San Jose, CA, USA). The activity was expressed as nmol of γ-GC produced per minute, per milligram of protein.

Measurement of Glutathione Levels

The intracellular levels of reduced and oxidized glutathione (GSH and GSSG, respectively) were determined using a fluorometric assay [25] with some minor modifications, in 96 multiwell UV plates. GSH levels were measured in 20 µg sample protein after the addition of 50 µg/mL o-phthalaldehyde (Sigma-Aldrich, St. Louis, MO, USA) and phosphate buffer (100 mM NaH_2PO4, containing 5 mM EDTA, pH 8.0) for 100 µL of final volume. After 15 min incubation, the end point fluorescence was measured using at 350 nm excitation and 420 nm. For determination of GSSG total levels, 25 µg protein was incubated for 30 min with 0.25 mg/mL N-ethylmaleimide (Sigma-Aldrich, St. Louis, MO, USA) in methanol and the final volume adjusted to 100 µL with 100 mM NaOH. The mixture was then incubated for 15 min with 50 µg/mL OPT in methanol and the fluorescence measured at 350 nm excitation and 420 nm emission using a Microplate Spectrofluorometer Gemini EM (Molecular Devices, San Jose, CA, USA).

Quantification of GPx and GRed Activities

Measurement of glutathione reductase (GRed) and glutathione peroxidase (GPx) activities was performed spectrophotometrically at 340 nm in 96 multiwell UV plates, through the analysis of NADPH oxidation, as described previously [25] with some minor modifications. For GPx activity determination, 15 µg of protein sample was mixed with 15 µL of potassium phosphate buffer 0.25 M containing 0.5 mM EDTA, pH 7.0 plus 1 mM GSH (Sigma-Aldrich, St. Louis, MO, USA) and 3,9 µL of GRed (SC 100-300 units/mg protein), the final volume adjusted to 150 µL with water and further incubated for 5 min at 30°C in the dark. The reaction was then started by addition of 0.25 mM NADPH (Sigma-Aldrich, St. Louis, MO, USA), 1.2 mM tert-butyl hydroperoxide and absorbance read for 5 min. For GRed activity measurement, 15 µg of sample protein was mixed with phosphate buffer (0.2 M K_2HPO_4 containing 2 mM EDTA, pH 7.0) and 0.2 mM NADPH and the final volume adjusted to 100 µL with water. The mixture was incubated for 30 sec at 30°C in the dark and the reaction started by the addition of 3 mM GSSG. GRed and GPx activities were determined using a SpectraMax Plus384 microplate spectrophotometer (Molecular Devices, San Jose, CA, USA).

Supplementary Data

A

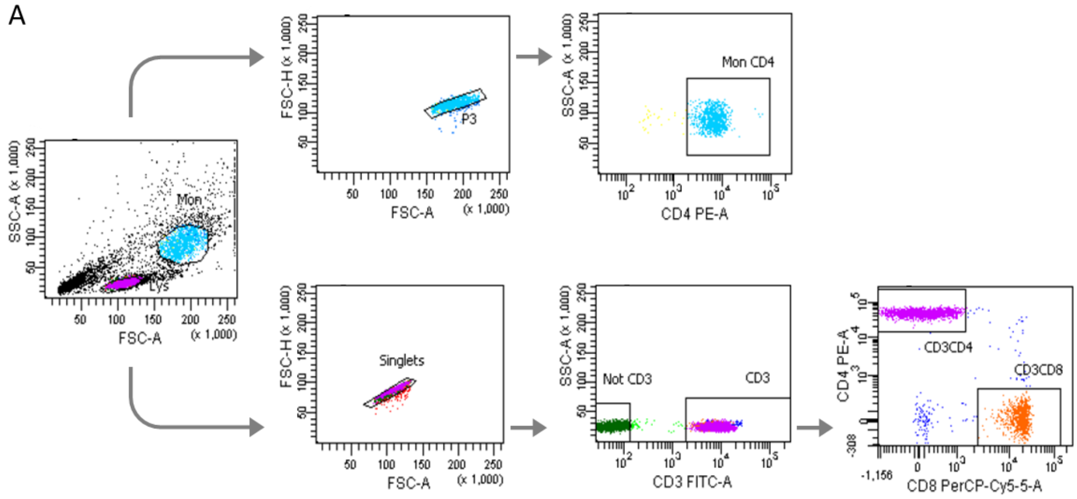

B

	Control	PDT	T2D	T2D-PDT
**CD3+CD8+**	18,62	17,09	15,76	13,57
**CD3+CD4+**	40,85	44,87	42,47	46,31
**Non CD3+**	30,83	28,40	34,63	29,18
**Monocytes**	9,69	9,64	7,14	10,94

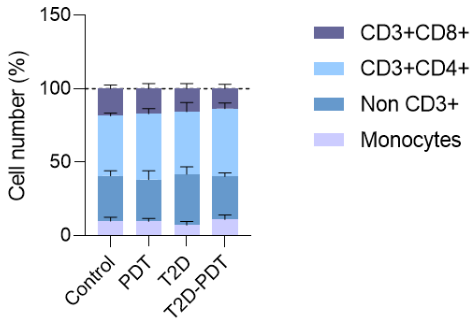

Figure S1. FACS separation of PBMCs subpopulations.

PBMCs derived from control individuals, PDT, T2D and T2D-PDT patients were sorted by FACS into CD3+CD8+ lymphocytes, CD3+CD4+ lymphocytes, non CD3+ lymphocytes and monocytes (A) and the number of cells plotted (B). Data are presented in percentage (%) of cells evaluated as the mean ± SEM.

Table S1. Characterization of experimental groups according to medication. Percentages reflect the number of subjects in a group taking a compound from the indicated class.

Subject	Alimentary Tract and Metabolism[1] *(antidiabetics)*	Nervous System[2] *(antidepressants + anxiolytics)*	Cardiovascular System[3]	Other Classes[4]
Controls	0%	50%	60%	50%
PDT	0%	10%	40%	50%
T2D	100%	30%	100%	50%
T2D-PDT	100%	30%	70%	40%

Medication nomenclature in accordance to the Anatomical Therapeutic Chemical (ATC) classification system and presented as subject percentage (%). [1]Gliclazide, Linagliptin, Metformin, Sitagliptin, Vildagliptin; [2]Alprazolam, Citalopram, Cloxazolam, Escitalopram, Mexazolam, Mirtazapine, Sertraline, Venlafaxine; [3]Acetylsalicylic acid, Amlodipine, Atenolol, Atorvastatin, Bisoprolol, Carvedilol, Cilazapril, Diltiazem, Enalapril, Etodolac, Fenofibrate, Fosinopril, Furosemide, Hidroclorotiazide, Indapamide, Irbesartan, Lisinopril, Losartan, Medoxomil, Nifedipine, Nimesulide, Nimodipine, Olmesartan, Perindopril, Pravastatin, Propanolol, Rosuvastatin, Sinvastatin, Telmisartan, Ticagrelor, Valsartan; [4]Alopurinol, Ascorbic acid, Etoricoxib, Calcitriol, Calcium, Ciclobenzaprine, Estradiol, Flutamide, Folic acid, Ferrous sulfate Furosemide, Gabapentin, Glucosamine, Hesperidin, Hidroxidine, Lansoprazol, Levothyroxine, Omeprazol, Pantoprazol, Pentoxifiline, Ruscus aculeatus, Sulfonate, Tizanidine, Trimetazidine. [PDT: periodontitis; T2D: type 2 diabetes; T2D-PDT: type 2 diabetes plus periodontitis].
